# Vitreous Mediators in Retinal Hypoxic Diseases

**DOI:** 10.1155/2013/935301

**Published:** 2013-01-10

**Authors:** Roberto dell'Omo, Francesco Semeraro, Giulio Bamonte, Francesco Cifariello, Mario R. Romano, Ciro Costagliola

**Affiliations:** ^1^Dipartimento di Scienze per la Salute, Università del Molise, Via Francesco de Sanctis, 86100 Campobasso, Italy; ^2^Dipartimento di Oftalmologia, Università di Brescia, 25121 Brescia, Italy; ^3^HagaZiekenhuis, 2545 Den Haag, The Netherlands

## Abstract

The causes of retinal hypoxia are many and varied. Under hypoxic conditions, a variety of soluble factors are secreted into the vitreous cavity including growth factors, cytokines, and chemokines. Cytokines, which usually serve as signals between neighboring cells, are involved in essentially every important biological process, including cell proliferation, inflammation, immunity, migration, fibrosis, tissue repair, and angiogenesis. Cytokines and chemokines are multifunctional mediators that can direct the recruitment of leukocytes to sites of inflammation, promote the process, enhance immune responses, and promote stem cell survival, development, and homeostasis. The modern particle-based flow cytometric analysis is more direct, stable and sensitive than the colorimetric readout of the conventional ELISA but, similar to ELISA, is influenced by vitreous hemorrhage, disruption of the blood-retina barrier, and high serum levels of a specific protein. Finding patterns in the expression of inflammatory cytokines specific to a particular disease can substantially contribute to the understanding of its basic mechanism and to the development of a targeted therapy.

## 1. Introduction

Oxygen supply of the retina is provided by a dual circulation. The photoreceptors and the greater portion of the outer plexiform layer receive nourishment from the choriocapillaris, whereas the inner retinal layers are supplied by the superficial and deep capillary plexuses formed by branches of the central artery of the retina. Inner retinal layers show highest sensitivity to hypoxic challenges [1], whereas outer retinal layers are more resistant to a hypoxic stress [2].

The causes of retinal hypoxia are many and varied. Systemic causes include the cardiovascular effects of chronic obstructive airways disease and the ocular ischemic syndrome associated with arterial obstructive conditions such as carotid artery stenosis [3], hyperviscosity syndromes, anemia, and trauma [4, 5]. Most common causes of local retinal hypoxia include retinal artery and vein occlusion, diabetic retinopathy (DR), retinal detachment, uveitis, and retinopathy of prematurity.

The retinal tissue is capable of inducing protective mechanisms such as glycolysis, angiogenesis, vasodilation, and erythropoiesis under hypoxic-ischemic conditions [6]. These mechanisms deemed of putative importance for limiting the damage are lost within hours of the hypoxic-ischemic insult following which cell death and tissue damage occur [7].

Under hypoxic conditions, a variety of soluble factors are secreted into the vitreous cavity including cytokines, chemokines, and growth factors.

Cytokines, which usually serve as signals between neighboring cells, are involved in essentially every important biological process, including cell proliferation, inflammation, immunity, migration, fibrosis, tissue repair, and angiogenesis [8].

Chemokines are multifunctional mediators that can direct the recruitment of leukocytes to sites of inflammation, enhance immune responses, and promote stem cell survival, development, and homeostasis [9].

Growth factors have been detected from ocular fluid of patients with diabetic retinopathy and other retinal disorders [10].

Finding patterns in the expression of inflammatory mediators specific to a particular disease can substantially contribute to the understanding of the basic mechanism of this disease and consequently to the development of a targeted therapy.

## 2. Dosage of Vitreous Mediators

Recently, a particle-based flow cytometric analysis method (PFCAM) has been established to overcome some of the intrinsic limitations of the conventional enzyme-linked immunosorbent assay (ELISA) and it has been used to analyze the vitreous inflammatory mediators by multiplex bead in patients with several vitreoretinal disorders [11–14].

The technology utilizes microspheres as the solid support for a conventional immunoassay, affinity assay, or DNA hybridization assay which are subsequently analyzed on a flow cytometer. Overall, the fluorescent readout of the flow cytometric assay is more direct, stable, and sensitive than the colorimetric readout of the ELISA. As the ELISA requires enzyme amplification, it is prone to variability and errors in the amount of amplification.

The sensitivity of the flow cytometric systems can be enhanced further by reducing the number of beads per test. This increases the ratio of cytokine to capture antibody in each test without reducing the potential signal strength of the assay (the number of capture antibodies per bead). PFCAMs are more reproducible than the ELISA which exhibits a significant variation between experiments and between plates within experiments. PFCAMs are also more accurate and reliable because the data are calculated from the mean of dozens of beads, each of which functions as an individual replicate. For many cytokines, the multiplexed and uniplexed PFCA assays are comparable suggesting that multiplexing does not significantly reduce the overall quality of the assay. In contrast, the conventional ELISA has limited capabilities. Finally, PFCAMs are much cheaper than ELISA when six or more cytokines are measured simultaneously.

Despite these advantages of PFCAM over ELISA, it should be kept in mind that, whatever the test used, there are some conditions which can alter the vitreous concentration of a certain protein, independently from the intraocular secretion of the protein itself.

For example, high serum levels of a specific protein could influence its intravitreous concentration. Similarly, the disruption of the blood-retina barrier produces an increase of proteins in the vitreous fluid. Finally, vitreous hemorrhage, which often occurs in conditions like proliferative diabetic retinopathy and vein occlusion, can produce an influx of serum proteins, such as growth factors, into the vitreous fluid.

## 3. Oxidative Stress

Oxidative stress, which may occur because of an imbalance between the production and the removal of reactive oxygen species (ROS), is considered to be a critical mediator in injury secondary to ischemic disorders.

Superoxide anion (O_2_^•−^) is one of the major ROS. The release of O_2_^•−^ in retinal ischemia was proven either directly by electron paramagnetic resonance or indirectly by showing diminished damage after the administration of antioxidant drugs such as EGB 761 extracted from *Ginkgo biloba*, vitamin E, mannitol, superoxide dismutase, and several other compounds [15–20].

The importance of O_2_^•−^ is also indicated by the fact that a manganese superoxide dismutase mimetic and transgenic manganese superoxide dismutase gene inhibited ischemia/reperfusion-induced retinal injury and diabetes-induced oxidative stress [21, 22]. ROS formed during oxidative stress can directly attack polyunsaturated fatty acids and initiate ROS chain reactions that result in lipid peroxidation in cellular membranes and a variety of oxidized products, including aldehydes, which are extremely reactive and can damage biological macromolecules. Injury can occur distal to the initial site of ROS attack because aldehydes are relatively long-lived compared with free radicals [23].

The resultant end products are well-known peroxidation markers of polyunsaturated fatty acids and are capable of inducing apoptosis in neuronal cells [24].

### 3.1. Nitric Oxide

Nitric oxide is synthesized by the enzyme NO synthase (NOS) from L-arginine. NOS exists in three isoforms: neuronal (nNOS) and endothelial (eNOS) which are constitutively expressed and inducible (iNOS). Enhanced nNOS, eNOS, and iNOS expressions have been reported in the retina in response to hypoxia [25]. Glial cells have been suggested to be the major cell types producer [26] but infiltrating leukocytes may also be an important source of iNOS production.

NO has been described to have neuroprotective and neurotoxic roles [27]. For example, NO produced by the eNOS isoform represents a protective response, since it produces vasodilatation and increased blood flow, maintaining retinal perfusion in hypoxic-ischemic conditions [28, 29].

However, besides these beneficial effects, eNOS is also involved in vascular-endothelial-growth-factor (VEGF-) induced vascular hyperpermeability [30].

NO production from nNOS and iNOS contributes to cytotoxicity resulting in cell death and axonal damage. Other than the generation of free radicals, a number of pathways such as N-methyl-D-aspartate-(NMDA-) mediated intracellular Ca^2+^ influx and CREB-mediated transcription of apoptotic proteins such as Bax, Bad, and Bcl–xl are triggered by NO resulting in neuronal death [31–33].

In retinal ischemia, RGCs death has been reported to be due to the involvement of iNOS as it has been observed that iNOS-positive leukocytes enter the ganglion cell layer and surround the RGCs and cause their degeneration. NO induces the proapoptotic cascade in hypoxic neural tissues by increasing phosphorylation of Bcl-2 [31]. Other mechanisms by which NO contributes to cytotoxicity may be peroxynitrite-mediated oxidative damage, DNA damage, and energy failure [34–36].

It has been shown that NO can react with the superoxide anion (O2–) to form peroxynitrite (OONO–) [37] which is neurotoxic. NO alone, even at high levels, has been reported as nontoxic to cortical neurons, but becomes neurotoxic after its reaction with O2– to form ONOO– [38]. *In vitro *studies have shown that the formation of OONO– increases the VEGF-induced permeability of retinal microvascular endothelial cells [39] and tissue damage through DNA damage reduced cellular antioxidant defenses and lipid peroxidation [40, 41].

A common target for peroxidation is polyunsaturated fatty acids (PUFAs) present in membrane phospholipids. Lipid peroxidation of retinal membrane PUFAs results in the loss of membrane function and structural integrity [42, 43].

For reasons that remain unclear, retinal endothelial cells seem particularly susceptible to peroxidation-induced injury, whereas pericytes, smooth muscle cells, and perivascular astrocytes are relatively resistant [44–47].

The retina is highly susceptible to lipid peroxidation since 20% of its dry weight is composed of lipids containing a high level of different PUFAs including docosahexaenoic acid (DHA; 22:6*ω*−3), arachidonic acid (AA; 20:4*ω*−6), and choline phosphoglyceride. Retinal vessels, in contrast to parenchyma, contain saturated fatty acids like stearic acid as well as unsaturated ones including AA and DHA, but the important DHA precursor, eicosapentaenoic acid (20:5*ω*−3), is not detected in retinal vessels.

A large body of evidence supports the idea that the increase in oxidative stress in retinal microvasculature is a key factor for the development of diabetic retinopathy [48–50].

A large body of evidence has also demonstrated an increase in reactive oxygen species and NO production in different tissues and cell types during diabetes, or after the exposure to high glucose [50, 51], which have been claimed to contribute to the vascular alterations observed in diabetic retinopathy. In fact, oxidative and nitrosative stress have been associated to the increase of apoptosis in retinal endothelial cells exposed to hyperglycemic conditions [49, 52–55].

It has been demonstrated that elevated glucose per se induces an increase in the levels of ROS in retinal endothelial cells [49].

### 3.2. Excitotoxicity

Glutamate, the excitatory neurotransmitter in the retina, is released by photoreceptors, bipolar cells, and ganglion cells and mediates the transfer of visual signals from the retina to the brain [56].

Augmented release of glutamate and its accumulation in extracellular spaces in hypoxic-ischemic conditions, leading to the activation of glutamate receptors, has been implicated in hypoxic/ischemic neuronal death [57, 58].

Glutamate exerts its action through ionotropic (aminomethyl-propionic-acid (AMPA) *N*-methyl-D-aspartate (NMDA), and kainate glutamate receptors) and metabotropic receptors [59, 60]. Glutamate receptor-mediated damage has been reported to occur in glaucoma, central, and branch retinal arterial and retinal vein occlusions resulting in the loss of retinal ganglion cells [61].

Neurotoxic effects of glutamate are reported to occur predominantly through the activation of ionotropic glutamate receptors (GluR). NMDA receptors are highly permeable to Ca^2+^ [62–65], their activation resulting in an increase in the intracellular calcium levels [61, 65–67].

Ca^2+^ overloadd has been reported to be a central event in neuronal death during ischemia [68, 69].

In fact, abnormal higher concentrations of calcium lead to inappropriate activation of enzymes such as proteases, nucleases, and lipases which are harmful to the cellular constituents and generate free radicals as well as cause mitochondrial failure which results in energy depletion and further free radical production [70].

Depolarization of neuronal membranes due to energy failure results in Ca^2+^ influx through the voltage-dependent Ca^2+^ channels followed by Ca^2+^-dependent glutamate release [71] which further increases the extracellular accumulation of glutamate. Activation of ionotropic glutamate receptors also results in influx of Na^+^ and Cl^−^ ions, inducing osmotic swelling. Glutamate acting via NMDA receptors activates nNOS [72] and the production of NO [73].

Glutamate-induced activation of AMPA and NMDA receptors has been shown to enhance the production of tumor ncrosis factor (TNF)-*α* [74–76] and interleukin-1*β* (IL-1*β*) [77] significantly.

Cooperation between glutamate receptors and inflammatory cytokines may be one of the mechanisms involved in cell damage.

Glutamate toxicity also results in glutathione depletion and oxidative stress [78]. Glutathione is a major cellular antioxidant which protects the cells against oxidative stress [79–81]. Increase in intracellular ROS in response to glutathione depletion has been reported in several studies [82]. Removal of excess glutamate from the extracellular space by glutamate transporters is crucial to terminate glutamate excitotoxicity. Glutamate transporters are responsible for the removal of glutamate from the extracellular fluid in the retina. It has been suggested that excess glutamate accumulation in the extracellular spaces may result from a failure of the glutamate transporters, such as GLAST, in the vicinity of RGCs [83]. Glutamate transporters have been described as necessary to prevent excitotoxic retinal damage and to synthesize glutathione and their deficiency has been reported to result in RGC degeneration [83].

### 3.3. Role of Inflammation

Hypoxia-ischemia is known to attract macrophages to hypoxic areas through expression of monocyte-chemoattractant-protein-(MCP-) 1. The hypoxia-activated macrophages and microglia, the immune effector cells in the retina, release TNF-*α* which has been reported as a triggering factor to activate the production of interleukin- (IL-) 8, VEGF, and MCP-1 in retinal vascular cells and/or glial cells adjacent to microvessels [84].

Several inflammatory molecules including intercellular-adhesion-molecule-(ICAM-) 1, TNF-*α*, IL-1, NOS, and COX-2 released by activated inflammatory cells and glial elements play a major role in the degeneration of retinal capillaries [85, 86]. Expression of adhesion molecules, intercellular adhesion molecule-(ICAM-) 1, and vascular-cellular-adhesion-molecule-(VCAM-) 1 on the endothelial cells facilitating leukocyte adhesion and infiltration into the areas of damage, has been reported to be induced by TNF-*α* and IL-1 [87–90]. ICAM-1 is important for establishing adhesion of leukocytes before their movement across the endothelium into the tissue [91]. IL-1 and TNF-*α* may also be involved in transcriptional activation of the iNOS gene [92, 93].

The proliferative diabetic retinopathy (PDR) retinal environment is characterized by the upregulation of iNOS, COX-2, ICAM-1, caspase 1, VEGF, nuclear factor kappa-light-chain-enhancer of activated B cells (NF-*κ*B), and increased production of NO, prostaglandin E2, and IL-1*β*, as well as increased permeability and leukostasis. Localized inflammation is responsible for capillary occlusion and degeneration leading to the ischemia-induced vasculogenesis, which results in DR [94].

Increased leukocyte adhesion (via ICAM1-CD18) to retinal vascular endothelium with resulting endothelial damage, breakdown of the blood retina barrier, capillary nonperfusion, and ischemia contribute to neovascularization. Inhibition of integrin *α*-4, which forms a part of very late antigen-4 (VLA-4) that binds to VCAM-1, decreases TNF-*α*, VEGF, NF-*κ*B and reduces leukocyte adhesion and vascular leakage [95]. Cytokines produced by inflammatory cells play a central role in the pathogenesis of PDR by promoting leucocyte-mediated damage to retinal vasculature [96].

Nicotinamide adenine dinucleotide phosphate (NADPH) oxidase, produced by neutrophils, is associated with leukocyte adhesion and vascular leakage in diabetic maculopathy and neovascularization. NADPH oxidase is a mediator of DR possibly by reducing peroxisome proliferator-activated-receptor- (PPAR-) *γ* and activating the NF-*κ*B pathway [97].

*In vitro*, apocynin and superoxide dismutase prevented suppression of PPAR-*γ* in bovine retinal endothelial cells treated with high glucose [97]. In this environment, the balance is shifted in favour of matrix metalloproteinases (MMPs) and away from their inhibitors, tissue inhibitor of matrix metalloproteinases (TIMPs). MMP-2 and MMP-9 actively degrade collagen IV, which is a major component of basement membranes, causing the extracellular matrix degradation needed for angiogenesis in DR.

## 4. Cytokines

### 4.1. Tumor Necrosis Factor *α*

Tumor necrosis factor *α* is an inflammatory mediator of neuronal death after ischemic injury in the brain and retina [98]. TNF-*α* is a member of the death-inducing ligand (DIL) family; it triggers the extrinsic pathway of apoptosis and acts through its two primary receptors, TNFR1 (p55) and TNFR2 (p75).

TNF-*α* was identified and isolated because of antiangiogenic activity; when injected into tumors, it causes tumor vessels to regress resulting in tumor necrosis [99].

So, it is quite clear that TNF-*α* has antiangiogenic effects, but it may also have proangiogenic effects in some situations. Despite its inhibition of endothelial cell proliferation *in vitro*, sustained release of TNF-*α* in cornea or injection of 105 units of recombinant TNF-*α* into the vitreous cavity of rabbits causes cellular infiltration and neovascularization (NV) in the cornea [100, 101], possibly by induced expression of other proangiogenic proteins such as interleukin-8, VEGF, and fibroblast-growth-factor- (FGF-) 2 [102]. In cultured vascular endothelial cells, TNF-*α* induces expression of VEGF receptor 2 and neuropilin-1 [103]. In mice, subcutaneous implantation of a pellet containing a low dose (0.01–1 ng) of murine recombinant TNF-*α* stimulated angiogenesis, while implantation of a pellet containing a high dose (1–5 *μ*g) inhibited angiogenesis demonstrating opposite effects depending upon the concentration [104].

This paradox may be explained in part by the ability of TNF-*α* to activate 2 intracellular signaling pathways in endothelial cells, one leading to apoptosis [105] and one that promotes survival and proliferation through the activation of nuclear factor-kappa B (NF-*κ*B) [106]. In addition, TNF-*α* recruits inflammatory cells, which stimulate neovascularization (NV) in some situations and inhibit it in others [107, 108].

Finally, the ability of TNF-*α* to induce expression of proangiogenic molecules can result in different effects depending upon the makeup of the local cell population and its response to TNF-*α*. Therefore, the effect of TNF-*α* in various tissues and disease processes is difficult to predict and must be determined by experimentation.

Increased levels of TNF-*α* have been demonstrated in proliferative retinopathies and in animal models of retinal NV [109–112]. These increased levels of TNF-*α* may be collaborating with VEGF to stimulate retinal NV. TNF-*α* may also contribute to the process in other ways. For instance, leukocytes have been shown to play a role in the pathogenesis of ischemic retinopathies and TNF-*α* is a chemoattractant for leukocytes [111].

TNF-*α* also causes breakdown of the blood-retinal barrier [112] which may be related to its stimulation of leukostasis, and therefore TNF-*α* may contribute to the excessive permeability seen in ischemic retinopathies.

In early diabetic retinopathy, there is an increased release of retinal inflammatory mediators including TNF-*α* 85, IL-1*β*, ICAM-1, and angiotensin II [113] along with activation of microglial cells [114]. A soluble TNF-*α* receptor-Fc hybrid, such as etanercept [115], is able to normalize vascular permeability and leukostasis; this suggests that TNF-*α* contributes to diabetic retinopathy, perhaps by preventing endothelial-cell damage from adhering leukocytes [86].

### 4.2. IL-1, IL-6, and IL-8

Patients with PDR have increased vitreal levels of IL-1 and TNF-*α*, which induce ICAM-1 expression [116]. Aqueous humour levels of IL-6 and VEGF correlate with respective levels in the vitreous and their concentrations increase with severity of disease [117]. Early stage DR is associated with elevated levels of serum CD105 (which is thought to be involved in vascular remodelling) and vitreal VEGF which then decrease through the course of disease progression to severe PDR [118]. IL-6 (T-cell activation), IL-8 (neutrophil chemotaxis), MCP-1, and VEGF levels are also significantly higher in the vitreous of patients with PDR [119]. IL-18, which induces macrophage activation via interferon-gamma, is raised in the sera of patients with diabetes mellitus type 2 and background DR [120]. In line with previous findings, it has been shown that intraocular production of IL-6 rather than IL-8 appears to be associated with the neovascularization activity in PDR [117], although a significant linear correlation between IL-6 and IL-8 has been demonstrated [121]. However, severity grade of PDR is not related to either IL-6 or IL-8 expression levels in vitreous fluid. This reveals that increased IL-6 levels in vitreous might be a master regulator and an important clinical marker for neovascularization activity. In addition, IL-8 expression seems to be differentially regulated compared with IL-6 response in PDR process. Thus, interleukins play an important role in mediating the inflammation and neovascularization in the development of PDR.

### 4.3. High Mobility Group Box-1

High-mobility group box-1 (HMGB1) protein was originally described 30 years ago as a nonhistone DNA-binding protein [122], involved in nucleosome stabilization and gene transcription [123]. HMGB1 is expressed in ganglion cells layer, inner nuclear layer, outer nuclear layer, the inner and outer segments of photoreceptors, and in the retinal pigment epithelial cells in normal retina [124, 125].

In addition to advanced glycation end products (AGEs), HMGB1 is another ligand of the receptor for AGEs [126], which can contribute to the accelerated micro- and macrovasculopathy observed in diabetes [127]. However, HMGB1 may play a key role in the protection of retinal injury after ischemia-reperfusion [128] and is also implicated as an important endogenous danger signalling molecule amplifying the activities of immunostimulatory molecules in a synergistic manner [129, 130].

HMGB1 stimulates membrane ruffling and repair of a mechanically wounded endothelial cell monolayer, causes endothelial cell sprouting, and stimulates neovascularization of chicken embryo chorioallantoic membrane via RAGE [131]. The crucial role of HMGB1 has also been demonstrated in diabetic mice for ischemia-induced angiogenesis through a VEGF-dependent mechanism [132].

HMGB1 might play a role in the upregulation of VEGF-A in retinal ganglion cells after exposure to AGEs. It has been demonstrated that blocking HMGB1 with glycyrrhizin successfully inhibits AGE-BSA-induced upregulation of VEGF-A [133].

Therefore, HMGB1 works as a cytokine or a cofactor that amplifies the effect of the AGE-RAGE axis, in an autocrine/paracrine manner, and mediates the secretion of survival factors including VEGF-A for counteracting the oxidative stress.

## 5. Chemokines

Chemokines are multifunctional mediators that can direct the recruitment of leukocytes to sites of inflammation, promote inflammation, enhance immune responses, and promote stem cell survival, development, and homeostasis.

They are classified by structure into four groups, designated C, CC, CXC, and CX3C depending on the number and spacing of the cysteine residues in the mature protein.

The CXC chemokines are divided into two subgroups depending on the presence or absence of the sequence glutamic acid-leucine-arginine (ELR) that immediately precedes the first cysteine amino acid in the primary structure of these cytokines. The ELR-containing CXC chemokines are angiogenic. Most non-ELR CXC chemokines such as interferon-c-inducible protein of 10 KDa (CXCL10/IP-10) potently chemoattract activated T lymphocytes and are angiostatic [134].

### 5.1. Monocyte Chemotactic Protein-1

Abu El-Asrar et al. [135] demonstrated that in the vitreous humor of eyes with proliferative vitreoretinal disorders, the CC MCP-1 and the CXC chemokine IP-10 are detected at high levels not correlating to serum levels, suggesting an increased local production. Furthermore myofibroblasts in PDR and proliferative vitreoretinopathy membranes express MCP-1 and stromal-cell-derived-factor- (SDF-) 1, and vascular endothelial cells in PDR membranes express MCP-1, SDF-1, and the chemokine receptor CXCR3. The same authors found that MCP-1 levels in the vitreous from cases of active PDR are significantly higher than those in inactive PDR cases.

Collectively these findings provide evidence that increased MCP-1 expression contributes to the development of neovascularization and fibrosis in proliferative vitreo-retinal disorders.

Furthermore Hong et al. [136] showed that MCP-1 induces VEGF expression in endothelial cells; therefore, a positive regulatory feedback loop between VEGF and MCP-1 expression by vascular endothelial cells in mediating angiogenesis might exist.

### 5.2. Fractalkine

Fractalkine (FKN), the sole member of the CX3C chemokine family, is named for its fractal geometry. Silverman et al. demonstrated the presence of FKN in normal cultured microvascular endothelial and stromal cells of the iris and retina *in vitro* [137].

Vitreous sample from patients with PDR revealed higher FKN concentrations compared with the control and immunodepletion of soluble FKN from PDR vitreous samples caused 36.6% less migration of bovine retinal capillary endothelial cells [138].

Therefore, FKN appears, to be a potent angiogenic mediator *in vitro* and *in vivo* and may play an important role in ocular angiogenic disorders such as PDR.

### 5.3. Monokine Induced by Interferon-*γ*

Monokine induced by interferon-*γ* (Mig) is principally known as a chemoattractant of activated T cells, but also has an angiostatic activity. Wakabayashi et al. [139] have recently documented a significant elevation of vitreous Mig concentration in DR patients compared with control subjects. The authors also found a significant correlation between vitreous concentrations of Mig and VEGF. It is not clear why Mig, an angiostatic factor, is elevated in the vitreous in DR, where angiogenesis is one of the main pathologies. One possibility is that Mig is elevated as a response to the upregulation of angiogenic factors such as VEGF. A second hypothesis is that Mig in DR might be related to chemotaxis of leukocytes rather than to angiostatic functions, because leukostasis is considered one of the pathogenic mechanisms of DR [140].

### 5.4. Stromal Cell-Derived Factor-1

Stromal cell-derived factor-1 (SDF-1/CXCL12) is a member of the CXC chemokine family that was originally isolated from murine bone marrow stromal cells. CXCR4, a 7-transmembrane-spanning G protein-coupled receptor, is one of the two receptors for SDF-1.

Recent studies have shown that SDF-1/CXCR4 interaction plays an important role in endothelial progenitor cells (EPCc) migration differentiation, proliferation, and survival [141–145]. SDF-1 is upregulated in ischaemic tissues, establishing an SDF-1 gradient favouring recruitment of EPCs from peripheral blood to sites of ischaemia, thereby contributing to accelerated neovascularization [141, 142].

In addition, SDF-1 promotes the chemotaxis of bone-marrow-derived CD34+ stem cells and their differentiation into EPCs in ischaemic tissue and tumours [142, 144, 145]. CXCR4 blockade profoundly inhibits VEGF- and SDF-1-induced migration of EPCs and impairs incorporation of EPCs into sites of ischaemia-induced neovascularization [143].

The finding that VEGF-mediated migration of EPCs was also influenced by CXCR4 antibodies points toward a more general involvement of CXCR4 and its downstream signalling in the homing mechanisms of EPCs.

Butler et al. [146] reported increased SDF-1 levels in vitreous from patients with PDR. In a murine model of retinal ischaemia, upregulation of SDF-1 and CXCR4 was detected in ischaemic retinas. A substantial amount of the increase in CXCR4 was caused by influx of CXCR4-expressing bone-marrow-derived cells. Pharmacological blockade of CXCR4 suppressed ischaemia- and VEGF-induced retinal neovascularization.

In a murine model of proliferative retinopathy, Blom et al. [147] demonstrated that intravitreal injection of blocking antibodies to SDF-1 prevented retinal neovascularization, even in the presence of VEGF.

Recently, it has been demonstrated that stromal CXCR4+ CD34+ cells are closely associated with the new vessels within the epiretinal membranes in eyes with PDR [148].

## 6. Transcriptional Factors

### 6.1. Hypoxia-Inducible Factor

Hypoxia-inducible-factor- (HIF-) 1 is a transcription factor that plays an essential role in the systemic homeostasis response to hypoxia. HIF-1 controls the expression of most genes involved in adapting to hypoxic conditions. HIF-1 triggers the activation of several genes that result in the production of VEGF and other angiogenic factors [149–153].

Several researchers have shown that diabetic factors result in HIF-1 production and angiogenesis. Treins et al. [154] have shown that insulin growth factor 1 stimulates accumulation of HIF-1 in human retinal pigment epithelial cells.

VEGF expression seems to be regulated through dual interdependent mechanisms. One involves HIF-1 directly and the other indirectly through NF-kappa B-mediated COX-2 expression and prostaglandin E2 production. Acute intensive insulin therapy exacerbates diabetic bloodretinal barrier breakdown through HIF-1 and VEGF [155].

This could explain why intensive control can result in transient worsening of diabetic retinopathy.

Recently, the presence of HIF-1a in the diabetic membranes has been shown [156]. HIF-1 is found more often and more intensely in diabetic preretinal membranes compared with nondiabetic idiopathic epiretinal membranes [157].

### 6.2. Nuclear Factor (NF)-*κ*B

NF-*κ*B is an ubiquitous inducible transcription factor that is a master regulator of immune responses, cellular proliferation, and apoptosis. NF-*κ*B is activated under hypoxic conditions and in retinal endothelial cells and pericytes exposed to hyperglycaemia *in vitro* and *in vivo*.

Frede et al. reported for the first time the role of NF-*κ*B in controlling HIF-1 gene expression in response to inflammatory stimuli [158]. Later on, a binding site has been identified for NF-*κ*B within the HIF-1*α* promoter [159]. Hypoxia has been shown to result in the activation of NF-*κ*B that subsequently can bind to the HIF-1*α* promoter. It is evident that both transcription factors might be crucial regulators for IL-6 and IL-8 expression in vitreous of PDR patients. In contrast to that hypothesis some authors [121] could not detect either NF-*κ*B or HIF-1*α* activity in vitreous samples isolated from patients with PDR. However, this does not exclude locally increased NF-*κ*B or HIF-1*α* activity, as it has been previously documented [160]. Locally increased NF-*κ*B or HIF-1*α* activity may be covered for their total transcription factor levels in vitreous extracts. In addition, there might be a periodic regulation of NF-*κ*B or HIF-1*α* activity in different hypoxic conditions [161, 162]. To date, the role of NF-*κ*B or HIF-1*α* in the regulation of PDR process is weakly understood.

## 7. Growth Factors

### 7.1. Vascular Endothelial Growth Factor (VEGF)

The VEGF family forms a part of the platelet-derived growth factor (PDGF) supergene family members which comprise four major and five minor isoforms: VEGF 121, VEGF 165, VEGF 189, and VEGF 206; VEGF 145, VEGF 148, VEGF 162, VEGF 165b (an inhibitory isoform binding to VEGFR-2), and VEGF 183. These isoforms derive from alternative exon splicing of the VEGF-A gene, located on chromosome 6p21.3 [163, 164] and are classified by their amino acids number. These cytokines bind to cell-surface receptors that belong to the family of tyrosine-kinase receptors [165].

VEGF binds to tyrosine-kinase receptors, VEGFR1 (Flt-1) and VEGFR2 (Flk-1), and also to the neurophilins (NP)-1 and 2, which also function as receptors. VEGF signalling is modulated by angiopoietins that bind to Tie-2 receptors [166].

VEGF-A has been studied extensively and plays a critical role in both vasculogenesis and angiogenesis [164, 167, 168].

The function of VEGF-A isoforms can vary during ocular development. It is generally accepted that in adults the formation of new blood vessels results exclusively from surrounding preexisting vessels by sprouting, a process referred to as angiogenesis whereas vasculogenesis, defined as the recruitment and *in situ* differentiation of vascular endothelial cells from circulating bone-marrow-derived endothelial precursor cells, is normally thought to occur only in the embryonic phases of vascular development.

In addition to angiogenesis and vasculogenesis, VEGF-A may participate in the maintenance of some vascular systems in the adult, but little is known of the role of VEGF-A in the maintenance of adult ocular vasculature. Different studies have shown that VEGF has a role in endothelial reparation after damage [169, 170].

VEGF is secreted by macrophages, T cells, retinal pigment epithelial (RPE) cells, astrocytes, pericytes, and smooth muscle cells in response to hypoxic and inflammatory stimuli. VEGF secretion is inducible by hypoxia-ischemia *in vitro* and *in vivo*, via hypoxia-inducible-factor*-* (HIF-) 1 dependent transcriptional activation [171]. A 3–12-fold increase in VEGF gene expression has been reported in hypoxia [172–174]. VEGF enhances the adhesion of leukocytes to vascular walls and increases ICAM-1 and VCAM-1 expression in the brain and retina [175–177].

In the eye, ischemic retinopathies such as PDR and retinopathy of prematurity are pathologic events which, through retinal capillary obliteration, promotes retinal ischemia.

Several reports suggest that VEGF is the critical proangiogenic cytokine [178, 179] and that a direct correlation clearly exists between intraocular VEGF levels and ischemic ocular neovascularization [180–182].

Increased production of VEGF and enhanced permeability of blood retinal barrier has been reported in the hypoxic retina and inhibition of VEGF production with melatonin reduces blood retinal barrier permeability [183].

VEGF is involved in retinopathy of prematurity, DR, and age-related macular degeneration, the leading causes of irreversible visual loss in developed countries from infants to the elderly [184].

### 7.2. Connective Tissue Growth Factor (CTGF)

Connective tissue growth factor (CTGF) is a 38 kD cysteine rich heparin-binding protein and is involved in stimulation of proliferation, angiogenesis, migration, extracellular matrix production, cell attachment, cell survival, and apoptosis [147].

CTGF has been proposed to play an important role in tubule-interstitial fibrosis as one of the major mediators of TGF-*β*. It has been shown to be hypoxia-inducible in human breast cancer cells [185].

However, the precise signalling mechanisms of the hypoxia-induced expression of CTGF remain unclear. CTGF is expressed in vascular beds and acts on multiple cell types. It is important for vessel growth during early retinal development and promotes the fibrovascular reaction in murine retinal ischemia after laser injury [186]. CTGF overproduction is proposed to play a major role in pathways that lead to fibrosis [187] in the vitreous of PDR patients.

The vitreous of PDR patients has elevated levels of both CTGF and VEGF and the ratio between CTGF and VEGF levels dictates the degree of fibrosis and angiogenesis. Raised CTGF levels are associated with VEGF and fibrosis, but only VEGF itself is responsible for neovascularization (NV) in PDR. *In vitro*, CTGF induced production of fibronectin and VEGF expression had no direct effects on vascular endothelial cells. CTGF may promote formation of proliferative membranes in PDR but not its cicatrization. It may be implicated indirectly in modulating VEGF expression but has no effects on retinal NV [188]. Anti-VEGF therapy can temporarily tip the CTGF/VEGF ratio towards a profibrotic environment [189].

### 7.3. Stem Cell Factor (SCF)

Stem cell factor (SCF), or kit ligand, is a peptide growth factor that exists as a membrane-bound protein but may be cleaved by proteases, such as matrix metalloproteinase-9 (MMP-9), to produce a soluble cytokine [190, 191].

SCF is important for the survival and differentiation of hematopoietic stem cells. The receptor for SCF, the proto-oncogene c-kit, is a tyrosine kinase that is expressed by bone-marrow-derived endothelial stem/progenitor cells [192, 193].

SCF ligand binding leads to phosphorylation and activation of the c-kit receptor and its downstream signaling proteins, which have been implicated in cell proliferation, cell adhesion and cell survival as well as chemotaxis [194–196].

Several studies have demonstrated that SCF/c-kit signaling promotes the survival, migration differentiation, and capillary tube formation of endothelial cells and plays an important role in ischemia-induced neovascularization [190, 192, 194, 196–198].

Abu El-Asrar et al. [199] demonstrated that (1) PDR membranes show immunoreactivity for SCF, c-kit, G-CSF, eNOS, and CXCR4 in vascular endothelial cells; (2) stromal cells expressed SCF, c-kit, eNOS, and CXCR4; (3) c-kit+ cells coexpressed the chemokine receptor CXCR4 and eNOS; (4) the number of blood vessels expressing CD34, c-kit, G-CSF, eNOS, and CXCR4 and the number of stromal cells expressing c-kit, SCF, eNOS, and CXCR4 in membranes from patients with active PDR were significantly higher than those in membranes from patients with inactive PDR; and (5) there were significant correlations between the number of blood vessels expressing the panendothelial marker CD34 and the number of blood vessels expressing SCF, G-CSF, eNOS, and CXCR4 and the number of stromal cells expressing SCF. These data support the notion that bone-marrow-derived cells contribute to neovascularization in PDR epiretinal membranes and that SCF/c-kit signaling may play a role in the pathogenesis of PDR.

### 7.4. Insuline-Like Growth Factor (IGF-1)

IGF-1 is produced locally in the human eye by a variety of cells including RPE cells, retinal capillary pericytes, endothelial cells, Muller cells, and ganglion cells. In cultured human RPE cells, IGF-1 is thought to exert its effect by inducing a dose-dependent increase in IGF- 1R phosphorylation and in VEGF mRNA levels. IGF-I1 also stimulates VEGF promoter activity *in vitro*, mainly via HIF-1alpha and secondarily via NF-*κ*B and AP-1 [200]. In a south Indian cohort, a CA 18-repeat genotype in the promoter of IGF-1 is implicated in susceptibility to PDR and associated with clinical severity [201].

### 7.5. Fibroblast-Growth-Factor- (FGF-) 2

Fibroblast growth factor (FGF)-2 is quickly released during the wound-healing process, providing an early stimulus for endothelial cell proliferation in the acute phase immediately after injury. FGF-2 appears able to upregulate VEGF production and acts synergistically in stimulating angiogenesis-platelet-derived growth factor, transforming growth factor-3.

### 7.6. Erythropoietin (Epo)

Erythropoietin, a stimulator of red blood cells, is also a promoter of vascular endothelial cell proliferation and angiogenesis [202]. Both Epo and VEGF respond to hypoxia [203] leading to ischemia-induced angiogenesis. Epo and VEGF are both raised in the vitreous of patients with PDR and act independently of each other [204].

Epo levels are higher than that of VEGF and its inhibition suppresses retinal NV both *in vivo *and *in vitro*. Suppression of Epo and VEGF leads to a greater inhibition of retinal NV than when either is inhibited alone. *In vitro *inhibition of Epo leads to attenuation of endothelial cell proliferation in PDR [205]. In murine models of oxygen-induced retinopathy, inhibition of Epo led to inhibition of retinal NV *in vivo *and inhibition of retinal endothelial cell proliferation *in vitro *[204]. Even though this evidence may tempt us to target Epo in the development of a retinal antiangiogenic strategy, we must be cognizant of its neuroprotective effects on retinal cells [206].

## 8. The Renin-Angiotensin (RAS) System

Human retinas have angiotensin receptor (ATR) type-1 and ATR-2. In human models of DR and hypoxia-induced retinal angiogenesis, the RAS is upregulated leading to the production of VEGF, PDGF, and CTGF leading to microvascular complications, angiogenesis, cell proliferation, and fibrosis [207].

The RAS exerts its effects by the generation of a family of bioactive angiotensin peptides among which angiotensin II (ANG II) and the ATR-1 and ATR-2 receptors are most well characterized [207]. Emerging evidence suggests that an ocular RAS is activated in DR and may contribute to progressive alterations to retinal cells such as pericytes, endothelial cells, neurons, and glia. In the kallikrein-kinin system (KKS), bradykinin (BK) and kallidin and their carboxypeptidase metabolites, des-Arg (9)-BK and des-Arg(10)-kallidin, are the effector peptides exerting their actions via BK type 1 (BK-B1) and BK type 2 (BK-B2) receptors. Both RAS and KKS damage the retinal vasculature and glia in DR via production of VEGF and CTGF [207]. The RAS is also implicated in progression of DR via Ang II. Ang II induces VEGF, which leads to the loss of tight junction proteins causing a breach in the integrity of the BRB. Angiotensin receptor blockers that block Ang II receptors reduce VEGF production by retinal endothelial cells and promote the recovery of tight junction proteins thus preventing progression of DR in its early stages [208]. Important cross-talk exists between the RAS system, advanced glycation end products (AGEs), and their receptors (RAGE). AGEs act via RAGE to cause diabetic microvascular complications leading to PDR [209]. *CCN1/Cyr61* is a member of the cysteine-rich 61/connective tissue growth factor/nephroblastoma overexpressed (CCN) family of genes. It is a downstream effector of AGE in the diabetic retina and may work synergistically with VEGF to cause ocular angiogenesis and PDR in models of oxygen induced retinopathy (OIR) in mice and streptozotocin (STZ-) induced DM in rats. Levels of both *CCN1 *mRNA and protein are raised in vitreous of STZ rats and PDR patients (nondiabetics) [210]. AGEs-RAGE-induced VEGF expression is thought to lead to neovascularization in PDR. Olmesartan, an angiotensin II type-1 receptor blocker, inhibited angiogenesis by inhibiting AGE-induced NFK-b promoter activity and consequently NFKb-mediated RAGE expression [211]. AGEs also induce injury of retinal pericytes, which are protected by PEDF expression. Thus, a decrease in PEDF expression can amplify the effect of AGEs on RPE integrity leading to PDR [212].

## 9. Other Mediators

### 9.1. Periostin

Periostin is a secreted extracellular matrix (ECM) protein that is found in areas of normal fibrogenesis or pathologic fibrosis and that can directly interact with other ECM proteins such as fibronectin, tenascin-C, collagens I and V, and heparin. The high degree of structural and sequence homology of periostin with fasciclin 1 and transforming growth factor *β*-induced suggests that periostin plays a role in cell adhesion and migration [213].

Yoshida et al. [214] showed that the concentration of periostin in the vitreous of patients with PDR is significantly higher than that in the vitreous of patients without PDR and, differently from the concentration of VEGF or bFGF, it is significantly correlated with the presence of fibrovascular membranes (FVMs). The differences in the correlations between periostin and VEGF are probably because VEGF is upregulated in the retina at an earlier stage in response to ischemia before the development of FVMs [215, 216].

### 9.2. Apelin

Apelin was first identified as an endogenous ligand of the orphan G-protein-coupled receptor, APJ, from bovine stomach extracts in 1998 [217]. Apelin signaling has recently been identified as an important contributor to angiogenesis [218]. It is reported that both apelin messenger RNA and APJ messenger RNA are highly expressed in the vascular system, especially in endothelial cells [219, 220].

*In vitro*, apelin was found to stimulate the proliferation and migration of retinal endothelial cells and the vascular tube formation [221].

Apelin might contribute to the formation of FVMs during the development of PDR and apelin may not be directly regulated by VEGF. Consequently, apelin signalling could represent a new promising therapeutic target during pathologic neovascularization associated with PDR [222].

### 9.3. Adiponectin

Adiponectin (APN) is a polypeptide hormone produced exclusively in adipocytes and circulates at very high levels in the bloodstream. In experimental studies, APN has been shown to exert anti-inflammatory and antiatherosclerotic effects and to inhibit neointimal thickening and vascular smooth muscle cell proliferation in mechanically injured arteries. Plasma APN concentrations are decreased in obesity, insulin resistance, type 2 diabetes, coronary disease, and hypertension [223]. Several studies have indicated that APN possesses anti-inflammatory properties and thus may negatively modulate the process of atherogenesis [224]. The role of APN in the development of microvascular disease (such as diabetic retinopathy and nephropathy) is largely unknown.

In patients with PDR, aqueous humor levels of APN are significantly higher than those recorded in control subjects and tend to diminish after intravitreal bevacizumab [225, 226]. These increased APN levels may represent a local reparative response to endothelial dysfunction.

Circulating APN levels well correlate with blood inflammatory marker levels, being highest in the presence of chronic inflammatory diseases. This effect is mediated by a downregulation of a TNF-*α*, whose levels are chronically increased in Type 2 diabetes. These remarks underline the relationships with inflammatory background and clearly indicate the role of APN as an endogenous modulator of microvascular function and inflammation.
